# Application of digital-intelligent technologies in physical education: a systematic review

**DOI:** 10.3389/fpubh.2025.1626603

**Published:** 2025-07-24

**Authors:** Qiying Zhong, Jiajun Jiang, Wenting Bai, Zhihua Yin, Ziyuan Liao, Xiaohui Zhong

**Affiliations:** ^1^College of Physical Education and Health, East China Normal University, Shanghai, China; ^2^School of Physical Education and Health, Shanghai University of International Business and Economics, Shanghai, China; ^3^College of Physical Education, Guangdong University of Education, Guangzhou, Guangdong, China; ^4^School of Physical Education and Health, Longyan University, Longyan, Fujian, China

**Keywords:** digital-intelligent technologies, artificial intelligence, physical education, systematic review, wisdom education

## Abstract

**Background:**

Digital-intelligent technologies (DIT), positioned at the convergence of artificial intelligence and digital transformation, have become pivotal to the modernization of physical education (PE). Although widely discussed within the context of Wisdom Education, their specific applications in PE have not been systematically synthesized. This study aimed to systematically review the scope, educational impacts, and implementation challenges of DIT in PE.

**Methods:**

A systematic review was conducted on 85 empirical studies published between January 2014 and November 2024. Following PRISMA guidelines, the review examined application scenarios, technological architectures, educational outcomes, and implementation barriers.

**Results:**

Digital Intelligent technologies (DIT) have been adopted across all stages of PE, forming a closed-loop system that includes (a) intelligent instructional design, (b) real-time process visualization, and (c) data-driven evaluation and feedback. Key innovations involved adaptive learning platforms, virtual simulation tools, multimodal assessment systems, and health behavior monitoring. Challenges remain in algorithmic accuracy, data privacy, and unequal access to digital resources.

**Conclusion:**

Digital-intelligent technologies (DIT) have reshaped physical education by enabling more intelligent, personalized, and data-informed instructional practices. To fully realize their potential, future efforts must prioritize algorithmic advancement, ethical safeguards, and the promotion of digital equity.

## 1 Introduction

The convergence of digital technologies and artificial intelligence has led to the emergence of “digital-intelligent integration,” a key trend in the evolution of educational technologies that has driven significant transformations in education systems (1). In this context, educational paradigms shifted from technology-driven informatization to a phase of “wisdom education,” characterized by intelligent decision-making, personalized learning, and adaptive instruction. This transition reflected a closer alignment between technological innovation and pedagogical objectives, accompanied by a stronger emphasis on learner-centered design and support strategies (2, 3).

As a discipline integrating cognitive development with physical practice, physical education has undergone notable transformations driven by technological innovations. In recent years, emerging technologies such as wearable devices, big data platforms, motion recognition systems, and virtual reality (VR) have been increasingly applied to physical education instruction, athletic performance assessment, and student health monitoring. These technologies have partially addressed long-standing challenges in traditional physical education, including spatial and temporal constraints, delayed feedback, and unequal access to resources (4, 5). Unlike cognition-oriented disciplines, however, physical education remains inherently embodied, with learning processes rooted in physical participation and kinesthetic experience. This technological infusion presents critical opportunities for the advancement of wisdom-driven physical education (6). Therefore, effective integration of digital-intelligent technologies (DIT) into physical education requires not only technical functionality but also pedagogical alignment with content characteristics and embodied learning modalities. While several systematic reviews have examined artificial intelligence or virtual reality in physical education, most have focused on isolated technologies or specific instructional contexts, lacking a comprehensive synthesis of broader trends, practical effectiveness, and long-term educational implications (7).

Building on this foundation, the present review systematically synthesized recent practices and research developments related to DIT in physical education (8). The study aimed to explore how DIT is applied in educational contexts, assess its impact on teaching and learning, and examine the key challenges associated with its implementation. Through this analysis, the review provides a theoretical foundation and practical guidance to support the digital-intelligent transformation of physical education in both national and international settings.

## 2 Literature review

Digital-intelligent technology (DIT) represents an integrated innovation paradigm resulting from the convergence of artificial intelligence, the Internet of Things (IoT), big data, and other advanced technologies built upon digital foundations. It extends beyond simple tool use by emphasizing the creation of synergistic ecosystems for multi-intelligent technologies within educational environments (9). In education, DIT has propelled instructional models toward “wisdom education,” characterized by the generation of personalized learning pathways, dynamic optimization of teaching strategies, and improved efficiency in educational governance. In the context of embodied learning, physical education has seen the emergence of “Wisdom Physical Education” (WPE) through digital-intelligent integration (10), marked by real-time data collection and analysis to develop personalized training programs, thereby advancing intelligent transformation across the full cycle of teaching, learning, practice, assessment, and management.

Digital-intelligent technologies have been comprehensively integrated into the physical education process through the synergistic application of wearable devices, big data analytics systems, and large AI models (5). Physical educators used wearable devices to collect students’ movement data, which were then analyzed using machine learning algorithms. Generative AI was further employed to construct learner profiles and develop personalized exercise plans, enabling data-informed instructional decision-making (11). This approach not only improved teaching efficiency but also promoted students’ self-awareness of performance and self-regulation of motor behaviors (12).

Currently, researchers have not reached a unified understanding of the concept of “Wisdom Physical Education” (WPE). Most scholars have interpreted the integration of modern information technologies into physical education as “Artificial Intelligence Physical Education” (AIPE) (5, 7, 13). Existing review studies have shown that research has primarily focused on virtual reality applications or evaluated the effectiveness of artificial intelligence in instructional feedback and health monitoring. However, these investigations have predominantly adopted a technological instrumentalist perspective, often overlooking the alignment between technologies and key human actors—such as teachers, students, and their interactive networks (11, 13–18).

Consequently, this study focused on the alignment between digital-intelligent technologies and human actors in physical education contexts. It systematically examined the types of applications, implementation modes, and practical constraints of these technologies, discussed their developmental trajectories in physical education, and provided reference points for future theoretical development and empirical investigation.

## 3 Methods

### 3.1 Study design

This study adopted a systematic review methodology in accordance with the PRISMA (Preferred Reporting Items for Systematic Reviews and Meta-Analyses) guidelines (19). As this review is narrative and qualitative in nature, it was not registered in PROSPERO, which primarily tracks reviews with quantitative synthesis. It synthesized the application landscape, technological ecosystem, and practical implications of digital-intelligent technologies (DIT) in physical education, covering the period from January 2014 to November 2024. The review was guided by the following research questions:

*RQ1:* What characterized the application landscape of DIT in physical education?

*RQ2:* How was the technological ecosystem of DIT in physical education?

*RQ3:* What were the practical implications of implementing DIT in physical education?

As this review did not involve human participants or animal subjects and relied solely on previously published data, ethical approval was not required.

### 3.2 Database search

A structured Boolean search strategy was implemented across Web of Science, Scopus, EBSCO, ACM Digital Library, Taylor & Francis, and Wiley Online Library. The search was refined to include only open-access, English-language records categorized as “Articles.” Two major keyword clusters were developed: (1) DIT-related terms (e.g., “Artificial Intelligence,” “Machine Learning,” “ChatGPT,” “Virtual Reality,” “Knowledge Graph,” “Intelligent Tutoring System”), and (2) PE-related terms (e.g., “Physical Education,” “Physical Education Teaching,” “School Physical Education,” “Sports Study”).

To reduce false-positive results, Boolean operators such as AND and OR were strategically used to combine terms and ensure topic relevance. Specifically, intersecting DIT and PE terms using AND restricted the retrieval to records that explicitly addressed both domains, while OR expanded within each keyword category to capture terminological variations. The search was further limited to the title (TI) and abstract (AB) fields to improve precision and filter out loosely related results. The complete query syntax is available in Supplementary material 1.

Concurrently, the PECOS framework (Population, Exposure, Comparator, Outcomes, Study Design) was applied to define the inclusion criteria. The target population focused on K–12 physical education settings, with digital-intelligent technologies (DIT) as the exposure variable, and empirical research designs (e.g., experiments, surveys, case studies) as the eligible study types. Following the initial database screening, a snowball sampling strategy was employed through Google Scholar to capture potentially overlooked studies (20), resulting in 2,945 candidate publications.

**Figure 1:**
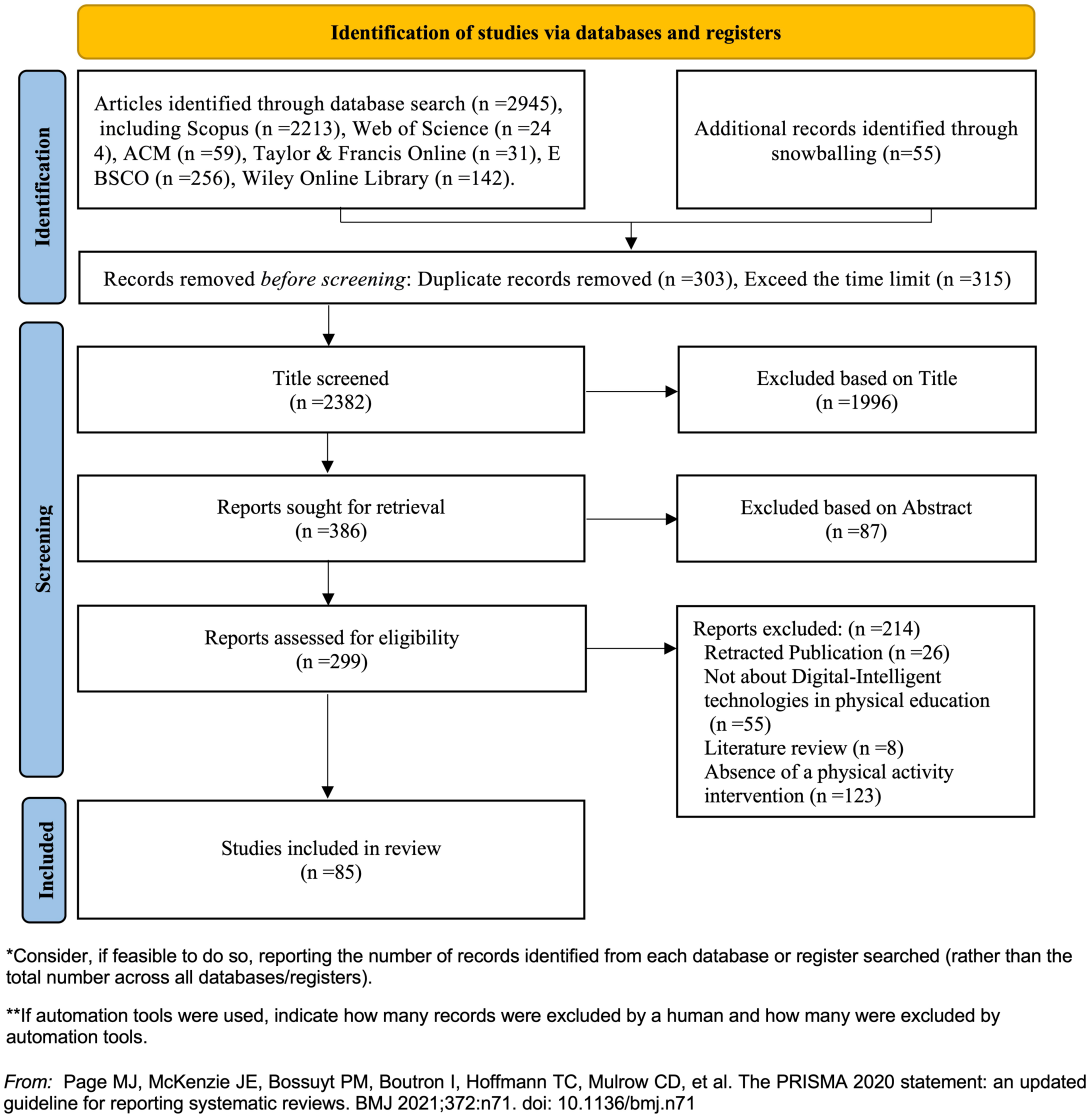
PRISMA flow chart of selection process.

Two independent reviewers (Qiying Zhong and Jiajun Jiang) independently conducted the full-text screening process. Inter-rater reliability, assessed via Cohen’s Kappa (*κ* = 0.82), indicated substantial agreement. Any discrepancies were resolved through structured deliberation. Qiying Zhong additionally conducted the initial title and abstract screening, and both reviewers jointly confirmed the eligibility of full-text articles (see Table 1). Ultimately, 85 articles satisfied the eligibility criteria and were included in the final analysis of this systematic review (see Figure 1).

**Table 1:** The inclusion and exclusion criteria.

Inclusion criteria	Exclusion criteria
Studies must be peer-reviewed, empirical investigations addressing the application of digital intelligent technologies in physical education.	Exclude non-empirical publications, including review articles, book chapters, conference proceedings, news reports, posters, and editorial materials.
Eligible study designs include randomized controlled trials (RCTs), pre-post intervention studies, crossover trials, and high-quality cross-sectional studies that clearly report PICO elements and measurable outcomes.	Exclude observational studies lacking PICO components or without quantitative outcomes.
Studies must be published in English between January 2014 and November 2024.	Exclude publications not in English or falling outside the specified timeframe.
Full-text availability must be ensured.	Exclude retracted publications and those inaccessible due to paywalls or missing data.
Studies must be published in peer-reviewed academic journals.	Gray literature, including dissertations, theses, preprints, and non-peer-reviewed online reports, is excluded to maintain quality control.

### 3.3 Data selection

The study employed a structured five-stage screening process to manage the retrieved literature: (a) Deduplication and temporal filtering: Duplicate records were removed, and publications outside the designated study timeframe were excluded; (b) Title screening: Literature was initially excluded based on irrelevance determined from the title; (c) Abstract screening: Abstracts were reviewed to eliminate studies that did not meet the inclusion criteria. As part of this process, articles not published in English were excluded to ensure analytical consistency and accessibility; (d) Full-text review: Two independent reviewers (Qiying Zhong and Jiajun Jiang) independently conducted the full-text screening process, they jointly confirmed the eligibility of full-text articles. Inter-rater reliability, assessed via Cohen’s Kappa (*κ* = 0.82), indicated substantial agreement; (e) Consensus resolution: Disagreements were resolved through discussion among the co-authors until consensus was reached (see Table 1). Ultimately, 85 articles satisfied the eligibility criteria and were included in the final analysis of this systematic review (see Figure 1).

### 3.4 Data analysis

This study employed bibliometric analysis (21), and qualitative content analysis (22) to extract and analyze data from the 85 included empirical studies. A classification framework was developed to categorize digital-intelligent technologies (DIT) application types in physical education, supported by representative examples. The framework was iteratively refined through discussions between two independent reviewers (Qiying Zhong and Jiajun Jiang) to ensure analytical consistency (23).

The data analysis process was independently conducted by Qiying Zhong and Jiajun Jiang to ensure analytical reliability. Data extraction utilized a standardized template including fields for study metadata (e.g., author, year, country), methodological characteristics (e.g., study design, sample size, type of Digital Intelligent Technology), functional, main findings, and quality appraisal indicators. The complete data extraction template is available in Supplementary material 2.

### 3.5 Risk of bias assessment

To ensure methodological rigor and evaluate the internal validity of the included studies, a structured risk of bias assessment was conducted using the Joanna Briggs Institute (JBI) critical appraisal tools, each aligned with the corresponding study design. Specifically, the JBI Critical Appraisal Checklist for Randomized Controlled Trials (24) was applied to studies utilizing random allocation; the JBI Revised Critical Appraisal Checklist for Quasi-Experimental Studies (2024 version) (25) was used for non-randomized intervention studies; and the JBI Critical Appraisal Checklist for Analytical Cross-Sectional Studies (26) was applied to observational studies with correlational designs.

Each appraisal item was rated using a dichotomous scoring system: a score of 1 was assigned if the criterion was clearly met (“Yes”), and 0 if it was not met or insufficiently reported (“No” or “Unclear”). The total quality score for each study was calculated by summing the individual item scores. The total quality score for each study was calculated by summing the individual item scores. For interpretability, studies were categorized into three quality levels based on their total scores: high (≥80%), moderate (60–79%), and low (<60%).

Two independent reviewers conducted the appraisals, and any discrepancies were resolved through structured discussion until full consensus was achieved. Although no studies were excluded solely on the basis of quality scores, the assessed risk of bias was systematically integrated into the narrative synthesis to contextualize the strengths and limitations of the available evidence. Detailed item-level results, scoring matrices, and final quality classifications are available in Supplementary material 3. Of the 85 studies included, 16 were rated as high quality, 68 as moderate, and 1 as low.

## 4 Results

The database searches initially identified 2,945 candidate records, including Scopus (*n* = 2,213), Web of Science (*n* = 244), EBSCO (*n* = 256), ACM Digital Library (*n* = 59), Taylor & Francis (*n* = 31), and Wiley Online Library (*n* = 142). An additional 55 records were retrieved through snowball sampling via Google Scholar. After preliminary screening, 303 duplicate entries and 315 records published outside the designated timeframe were excluded, resulting in 2,382 records for title screening. During the title and abstract screening phase, 2,083 studies were excluded due to irrelevance. A total of 299 articles were retrieved for full-text assessment. Ultimately, 85 studies met the inclusion criteria and were subjected to quality appraisal. These included 16 high-quality, 68 moderate-quality, and 1 low-quality study.

### 4.1 RQ1. Current application landscape of DIT in physical education

#### 4.1.1 Current research status and developments

**Figure 2:**
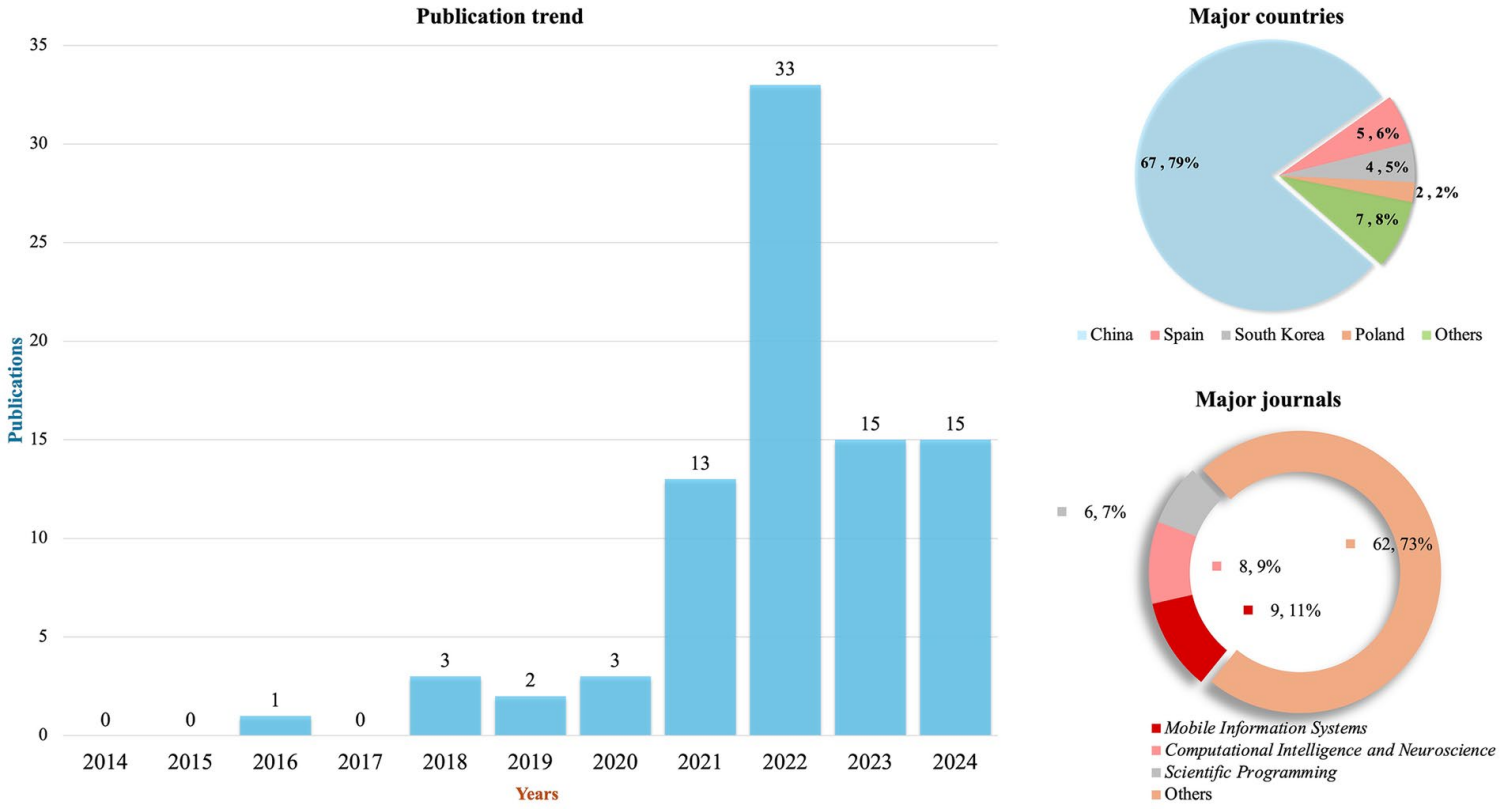
The publication situation of empirical research on the DIT in physical education.

Research on digital-intelligent technologies (DIT) in physical education has exhibited significant growth in recent years (see Figure 2). Since emerging as a focal topic in 2016, the number of empirical studies increased markedly between 2017 and 2020, reaching its peak in 2022—reflecting a surge in academic attention. Although publication volume stabilized after 2023, approximately 10 studies continue to be published annually, indicating sustained research interest and developmental potential in this evolving field.

Geographically, the 85 included studies originated from 11 countries or regions, with China contributing the majority (*n* = 67, 79%)—far surpassing other nations and reflecting increased research productivity driven by national digital education initiatives. Spain (*n* = 5, 6%), South Korea (*n* = 4, 5%), and Poland (*n* = 2, 2%) established recognizable research presences. In addition, Japan, the United States, Portugal, Malaysia, Oman, Vietnam, and Kazakhstan each contributed one study, highlighting the growing global engagement with digital-intelligent technologies in physical education.

Furthermore, the 85 included studies were published across 45 distinct academic journals, reflecting the diversity of dissemination pathways. *Mobile Information Systems* featured the highest number of publications (*n* = 9, 11%), followed by *Computational Intelligence and Neuroscience* (*n* = 8, 9%) and *Scientific Programming* (*n* = 6, 7%). These patterns highlight the strong cross-disciplinary appeal of digital-intelligent technologies within the domains of information science and intelligent computing.

#### 4.1.2 Application targets

The target populations of digital-intelligent technologies (DIT) applications in physical education exhibited clear stage-specific and group-based characteristics.

In terms of educational stages, the majority of studies focused on higher education (*n* = 68, 80%), which may reflect the advantages of universities in resource availability, curricular flexibility, and technological readiness. In contrast, relatively fewer studies addressed primary education (*n* = 11, 13%) and secondary education (*n* = 8, 9%), while only one study involved preschool settings (*n* = 1, 1%) (27), indicating the early-stage implementation of digital-intelligent technologies (DIT) in physical education for younger learners.

Regarding group-based focus, most studies investigated student populations (*n* = 60, 71%), emphasizing their central role in personalized learning, training feedback, and health promotion (28). Studies involving educators accounted for 29% (*n* = 25), primarily examining the potential of DIT to support instructional delivery and teacher professional development (29). Across all included studies, typically developing populations comprised 98% (*n* = 83), while only two studies addressed special populations (30, 31), highlighting a critical need for future research on DIT applications for students with disabilities, chronic health conditions, and other special educational needs in physical education.

#### 4.1.3 Application scenarios

Digital-intelligent technologies (DIT) applications in physical education were predominantly implemented in offline physical education courses, accounting for 86% (*n* = 73) of cases. These studies primarily leveraged intelligent devices, AI analytics, and big data monitoring to enhance teaching efficiency and students’ kinesthetic experiences (32). In contrast, the use of DIT in online physical education courses (*n* = 5, 6%) and blended physical education courses (*n* = 7, 8%) was comparatively limited. Despite the momentum generated by the COVID-19 pandemic in advancing digital education, integration depth and research coverage in these environments remained limited.

Regarding curricular contexts, DIT was predominantly applied within in-school courses (*n* = 81, 95%), with significantly fewer applications in after-school sports activities (*n* = 4, 5%). This distribution reflects an enduring research emphasis on formal instructional settings, while non-formal learning contexts and after-school sports engagement continue to be underexplored.

#### 4.1.4 Functional realization

The functional realization of digital-intelligent technologies (DIT) in physical education spans four key stages of the instructional cycle: curriculum planning, implementation, assessment, and feedback.

During curriculum planning, DIT facilitated intelligent scheduling of time–space resources and equipment allocation through intelligent curriculum systems (*n* = 7, 8%), reducing resource inefficiencies while also tracking student attendance and activity engagement (33).

The implementation phase demonstrated the most diverse applications, including intelligent teaching system (*n* = 35, 41%), auxiliary learning system (*n* = 21, 25%), and health risk monitoring (*n* = 7, 8%). Intelligent teaching system supported personalized training plans and incorporated VR to create immersive learning environments (34). Auxiliary learning system utilized multidimensional visual analytics to enhance kinesthetic immersion, promote engagement, and improve motor skill acquisition (35). Health surveillance tools employed wearable biosensors for real-time biomechanical monitoring to anticipate potential injury risks (36).

For automated teaching quality assessment (*n* = 8, 9%), DIT enabled automated performance evaluation using big data analytics and machine learning algorithms to conduct multidimensional analysis and generate instructional recommendations (37). In the feedback phase, the wisdom scheduling systems (*n* = 7, 8%) provided precise movement feedback through computer vision and AI systems that delivered real-time motion analysis and corrective guidance to improve athletic proficiency (38).

### 4.2 RQ2. The integrated framework of DIT in physical education

#### 4.2.1 Foundational algorithms

Algorithmic architectures form the operational backbone of digital-intelligent technologies (DIT), with machine learning (ML) algorithms playing a central role in enabling pattern recognition, behavioral prediction, and personalized recommendation through continuous training on multimodal datasets. Among the 85 included studies, 49 applied ML algorithms, of which 41 specified algorithm types—collectively identifying 21 commonly used algorithms across six taxonomic categories (see Table 2).

**Table 2:** Machine learning algorithms have been applied in Physical Education.

Algorithm	Principle	Application Scenario	*n*	Ref.
Unsupervised learning				
Spectral Clustering	Graph theory and feature decomposition	Data clustering	1	(37)
k-means	Distance-based partitioning		3	(70–72)
Canopy-K-means	Pre-cluster optimization		1	(73)
Deep Topological Clustering	Deep learning + topological structure		1	(33)
Supervised learning				
Support Vector Machine (SVM)	Maximizing the margin between classes	Classification tasks	6	(43, 74–78)
KNN	Voting based on nearest neighbors		2	(59, 78)
Probabilistic Neural Network (PNN)	Probability density estimation		1	(79)
Elastic Net	L1 + L2 regularization	Regression tasks	1	(53)
Sequence modeling				
Hidden Markov Model (HMM)	Hidden State Sequence Modeling	Sequence Prediction	3	(42, 51, 79)
RNN/LSTM	Recurrent Memory Unit	Sequence Processing	2	(27, 80)
Ensemble learning				
AdaBoost	Weak Classifier Integration	Classification	1	(53)
LightGBM	Gradient Boosting Tree	Classification/Regression	1	(81)
Random Forest	Decision Tree Ensemble		2	(82, 83)
Deep learning				
CNN/1D-CNN	Convolutional Feature Extraction	Image/Sequence Processing	7	(28, 40, 41, 77, 84–86)
BPNN/DNN	Multi-layer Perceptron	General Prediction	6	(42, 57, 87–90)
SOM	Competitive Learning Mechanism	Data Visualization	1	(91)
Hybrid model				
Dynamic Bayesian Hybrid Model	Dynamic Network + Hybrid Modeling	Multi-task Processing	1	(92)
Multifunctional Fuzzy Evaluation Model	Fuzzy Logic + AI	Complex Evaluation	1	(29)

Convolutional Neural Networks (CNNs) were the most frequently adopted, known for their ability to process grid-structured data and extract spatiotemporal features from dynamic movement imagery in physical education contexts (39). CNNs were often integrated with other technologies to enhance system functionality. For example, Guangde (40) combined CNNs with positioning systems and computer vision to develop a Deep Learning–based Physical Education and Emergency Response System (PEERS-DL), which used GPS-enabled monitoring to track athletic activities in real-time and achieved a peak risk detection accuracy of 97.61%. Similarly, Cao, Xiang et al. (41) introduced the Intelligent Physical Education Tracking System (IPETS), integrating big data analytics, VR, and intelligent recognition to evaluate students’ motion trajectories. Furthermore, Zong, Lipowski et al. (28) incorporated CNNs into IoT infrastructures to enable real-time classroom analytics, leading to a substantial improvement in students’ emotional regulation—from scores below 60 to above 79 on standardized metrics.

Furthermore, several studies applied ML algorithms in combination with optimization techniques and swarm intelligence to improve model stability and performance. For instance, Li (42) developed the Intelligent Behavioral State Parsing in PE (IBSPE) model using Backpropagation Neural Networks (BPNN) and Hidden Markov Models (HMM), which achieved behavioral state recognition accuracy exceeding 96%, offering predictive support for instructional goal setting. Li and Zhong (43) employed Hybrid Particle Swarm Optimization (HPSO) to refine a Firefly Algorithm–optimized Neural Network (FA-NN), significantly boosting prediction accuracy through metaheuristic optimization.

#### 4.2.2 Heterogeneous system integration patterns

Effective implementation of digital-intelligent technologies (DIT) in physical education requires the integration of multiple technological enablers. Based on an analysis of the 85 included empirical studies, this review identified three core technological clusters that underpin current DIT system architectures: the Human–Computer Interaction (HCI) Cluster, the Artificial Intelligence (AI) Cluster, and the Internet of Things (IoT) Integration Cluster. These clusters represent distinct but interrelated pathways for realizing wisdom educational environments and serve as foundational structures for subsequent technical development and pedagogical application.

First, The Human–Computer Interaction (HCI) Cluster centers on computer graphics technologies—particularly virtual and augmented reality (VR/AR)—and somatosensory interaction systems. These are frequently integrated with IoT sensor networks, big data analytics platforms, and machine learning algorithms to construct intelligent systems that support skill acquisition in physical education. Jiang, Wu et al. (12) designed an embedded sensor system specifically for basketball motion recognition, enabling the assessment of students’ fundamental basketball skills through motion analysis. Moreno-Guerrero, García et al. (44) implemented AR-based spatial orientation training, which significantly improved student engagement, academic performance, and technology acceptance. Liang, Zhang et al. (45) developed real-time 3D motion modules using the Unity 3D engine and instructional videos, further enhanced with tangible manipulatives aligned to curriculum content.

Second, The Artificial Intelligence (AI) Cluster centers on machine learning algorithms and their integration with multi-objective optimization, swarm intelligence, decision support systems (DSS), and big data analytics platforms. These technologies are primarily applied to instructional decision-making and learning assessment systems, enabling precise evaluation and predictive analysis of pedagogical effectiveness. For instance, Wang, Lima et al. (37) enhanced fuzzy evaluation methods by incorporating gray system theory with relational analysis, effectively reducing misclassification risks in kinematic data. Li, Wang et al. (29) integrated natural language processing and machine learning through fuzzy control rules to develop the Multi-feature Fuzzy Evaluation Model (MFEM-AI), which assessed technological proficiency in collegiate physical education. Similarly, Li (42) achieved dynamic behavioral state recognition and temporal classification using a combined BPNN–HMM architecture, resulting in motion recognition accuracy exceeding 96%.

Third, The Internet of Things (IoT) Cluster centers on 5G mobile communication networks and integrates software-defined networking (SDN), edge computing nodes, spatiotemporal big data platforms, and multimodal machine learning algorithms. This cluster emphasizes real-time data processing, distributed architectures, and high-level data interconnectivity, thereby supporting platform development and remote assessment in physical education. For instance, Wang (46) leveraged IoT architectures and wireless networks—combined with conventional campus infrastructure—to develop e-physical education platforms capable of visualizing instructional content and capturing real-time pedagogical activity data. Yao, Wang et al. (47) developed the IoT-based Technological Acceptance Learning Management Framework (IoT-TALMF), which achieved 97.33% identity verification accuracy, a 96.20% student performance ratio, and 97.12% system reliability. This system conducted statistical analysis of curricular content to define learning objectives and optimize resource allocation. Ning, Li et al. (11) built the Physical Education IoT Monitoring and Training System (PE-IoMT), in which cloud servers were used for data storage, analytics, and processing. The system comprehensively evaluated physical states and generated personalized training regimens designed to prevent sports injuries.

#### 4.2.3 Hierarchical support infrastructures

The functional realization of digital-intelligent technologies (DIT) in physical education critically depends on the integrated deployment of multiple intelligent systems (48). Based on a synthesis of the included studies, this review classified these support systems into six typologies: (a) intelligent curriculum management systems, (b) intelligent teaching systems, (c) auxiliary learning systems, (d) health risk monitoring systems, (e) automated teaching quality assessment systems, and (f) wisdom scheduling systems. Each category is described in detail below:

Intelligent curriculum management systems primarily support the administration of instructional and facility-related resources and can be categorized into two subtypes: (a) Sports Resource Assessment Indices (*n* = 3), and (b) Instructional Information Service Platforms (*n* = 4). For example, Hao and Zhou (49) developed an IoT-based Digital Twin System for sports facilities, which integrated edge computing and collaborative filtering algorithms to dynamically match user activity demands with venue resources, thereby improving facility utilization. Liu (50) designed a big data–enabled Instructional Information Platform that centralized the management of course registration, grading, and fitness assessment data, significantly enhancing processing efficiency and reducing teachers’ administrative burdens.

Intelligent teaching systems support human–AI collaboration and cognitive augmentation within classroom settings. This category includes four subtypes: (a) Educational Exergaming Systems (*n* = 2), (b) Wisdom Gymnasium Infrastructures (*n* = 3), (c) Pedagogical Robotics (*n* = 3), and (d) Interactive AI Tutoring Systems (*n* = 20). For example, Xu, Zhai et al. (51) developed a Kinect-based exergaming framework for children, enabling real-time motion trajectory extraction and motor skill assessment. The system incorporated adaptive learning path optimization algorithms that dynamically generated personalized intervention strategies based on learner performance metrics. When performance deficits were detected, the system triggered additional instructional scaffolding within the game environment. Traditional physical education is increasingly converging with modern e-learning modalities—including mobile internet, big data, and cloud computing—while being augmented by VR-enhanced interactive learning ecologies that demonstrably improve classroom interactivity and student engagement (35).

Auxiliary learning systems primarily operate within an Extended Reality (XR) framework, integrating multimodal human–computer interaction (HCI) technologies such as virtual reality (VR) and augmented reality (AR) to construct biomechanically grounded virtual simulations of three-dimensional human movement. Through immersive head-mounted displays (HMDs), haptic feedback gloves, and voice interaction modules, learners interact with motion-capture–driven 3D avatars for skill transfer training. These systems apply multimodal sensory integration to enhance cross-modal feedback across visual, auditory, and proprioceptive channels, significantly improving kinesthetic experiences and learning efficacy in physical education (36).

Health risk monitoring systems enable continuous tracking of students’ physiological responses to physical activity through wearable devices and IoT sensor networks. This system category comprises two subtypes: (a) Exercise Load Monitoring (*n* = 2) and (b) Biometric Data Surveillance (*n* = 4). These systems collect real-time physiological metrics—such as exercise intensity load, heart rate variability (HRV), gait characteristics, and locomotor distance—via wearable sensors. Built upon IoT frameworks, they facilitate cloud-synchronized transmission of multimodal data and integrate big data analytics with data mining techniques to develop predictive models for recovery optimization and injury risk assessment. Additionally, GPS-enabled geofencing monitoring is employed, with real-time biofeedback mechanisms delivering early injury alerts and supporting preventive interventions (28, 40).

Automated teaching quality assessment systems utilize deep convolutional neural network (CNN) architectures integrated with multi-source IoT sensor data to establish a Transfer Learning–based Educational Assessment Paradigm (TLEAP). This system architecture comprises three key components: (a) a Multimodal Data Perception Layer, which collects real-time instructional data from various sensory inputs; (b) an Enhanced ResNet Feature Extraction Module, which processes spatiotemporal teaching behavior patterns; and (c) a FAHP–GRA hybrid knowledge graph model (Fuzzy Analytic Hierarchy Process – Gray Relational Analysis), which enables effective mapping between expert knowledge and AI-derived feature spaces. Attention mechanisms are further employed to quantify correlations between instructional behaviors and quality evaluation metrics, thereby improving the system’s adaptability and real-time responsiveness (28, 37).

Wisdom scheduling systems encompass two subtypes: (a) Motor Skill Assessment Feedback Systems (*n* = 4) and (b) Athletic Performance Prediction Systems (*n* = 2). These systems typically employ decision tree algorithms integrated with machine learning, statistical analysis, intelligent databases, and neural network technologies. By mining data from contemporary physical education assessment databases, such algorithms generate latent evaluation models to support instructional administrators and policymakers (52). The constructed models draw on behavioral data collected in physical education classrooms—such as velocity, technical proficiency, and strength indicators—to enable real-time behavioral performance analytics and feedback generation (53).

### 4.3 RQ3. Implications and challenges of DIT in physical education

#### 4.3.1 Impacts

##### 4.3.1.1 Creating virtual athletic environments

The integration of digital-intelligent technologies (DIT) into physical education facilitates the construction of high-fidelity virtual reality (VR) training environments, immersing learners in interactive three-dimensional scenarios while supporting the formation of accurate motor schemas through multimodal sensory channels (54). The fusion of virtual environments and embodied physical experiences overcomes the spatial and temporal constraints of traditional pedagogy. Dynamic scene reconstruction addresses limitations such as equipment scarcity, training monotony, and outdoor safety risks (55). Human–computer interaction (HCI)-enabled intelligent collaboration systems allow geographically dispersed teachers and students to engage in synchronous skill demonstrations and receive real-time corrective feedback. Empirical findings by Wang, Abdul Rahman et al. (56) indicate that 80.0% of participants reported increased learning motivation, 75% demonstrated enhanced attentional engagement, and 63% experienced improved learning efficiency. By combining immersive kinesthetic experiences with biomechanically grounded feedback, such systems significantly enhance learners’ self-efficacy and attentional regulation during complex motor skill acquisition.

##### 4.3.1.2 Enhancing instructional decision-making capabilities

AI-driven automated teaching quality assessment systems, by integrating multisensory data, enable real-time monitoring and analysis of pedagogical processes. Embedded decision-support modules generate personalized instructional strategy recommendations. For example, Yang, Oh et al. (57) found that voice-interactive pedagogical robots in physical education enhanced teacher–student communication and supported real-time query resolution, leading to a 21-point increase in learning interest and a 9.8-point improvement in learning attitudes on standardized evaluation scales. In addition, intelligent motion demonstration units—utilizing 3D biomechanical deconstruction and standardized motion template matching—established visual reference frameworks for skill acquisition. Meanwhile, cloud computing and big data analytics were integrated with physiological sensors, multi-terminal resource distribution systems, and learning behavior visualization modules to develop a comprehensive instructional support platform. This unified platform enabled high-frequency teacher–student interaction through instant messaging and formative assessment tools, facilitating continuous calibration of learning objectives (57, 58).

##### 4.3.1.3 Enhancing physical-psychological wellbeing

Physical education fundamentally embodies somatic pedagogy, wherein the application of digital-intelligent technologies (DIT) ultimately aims to promote student health and wellbeing. Through the deep learning capabilities of augmented learning, feedback, and health monitoring systems, intelligent assessment mechanisms have been established that support bidirectional cognitive–behavioral regulation. Adaptive algorithms dynamically quantify students’ motor competencies and health conditions, generate personalized exercise prescriptions, and implement tiered interventions—effectively enhancing psychophysiological adaptation for individuals with exercise anxiety or motor impairments (59). Moreover, digitally reconstructed training environments with calibrated task difficulty allow students with participation barriers—such as low motivation, physical limitations, or skill deficiencies—to attain comparable learning outcomes to their motor-advantaged peers (16). These interventions are further supported by AR-based real-time achievement visualization systems, which significantly improve students’ kinesthetic self-awareness and sustain their engagement intentions. Collectively, DIT leverages multimodal human–computer interaction and big data analytics to construct immersive skill-acquisition ecologies tailored to digital natives, thereby achieving concurrent improvement in motor proficiency and overall health outcomes (38).

#### 4.3.2 Challenges

##### 4.3.2.1 Insufficient algorithm adaptability

Current intelligent physical education systems primarily rely on general-purpose educational models that lack specificity for the unique demands of sports contexts (60). For instance, existing algorithms for classroom attention monitoring and static behavior recognition perform inadequately when addressing the high-frequency, unstructured, and dynamic movement patterns characteristic of physical education settings. Within “Learning Feedback Systems” and “Augmented Learning Support Systems,” although multi-source perceptual data—such as the real-time fusion of Kinect skeletal tracking and inertial sensor inputs—are jointly analyzed, a robust modeling framework for cross-modal temporal alignment remains notably absent.

Moreover, algorithmic modeling of key variables—such as personalized exercise pathways, non-normative movement learning, and complex physiological load responses—is substantially underdeveloped. This limitation constrains critical functionalities such as early warning of sports injuries, adaptive management of feedback latency, and real-time generation of instructional strategies. Most current approaches still depend on rule-based matching to detect erroneous movements and have yet to establish highly reliable deep learning models with generalizability and temporal sensitivity (37). Insufficient algorithm adaptability thus represents a significant bottleneck, undermining system performance in context-specific adaptation, motion feedback precision, and real-time responsiveness.

##### 4.3.2.2 User data leakage risks

The application of digital intelligence technologies in sports education necessitated processing massive datasets encompassing students’ motor performance, physiological states, and spatial trajectories. Such data were typically acquired in real-time via IMU sensors and environmental collection devices, then uploaded to cloud platforms for processing and analysis. The core functionalities of “health risk monitoring systems” and “automated teaching quality assessment systems” were precisely built upon these high-frequency, multimodal datasets. However, most current systems had yet to establish comprehensive data governance mechanisms covering the entire lifecycle, exhibiting significant deficiencies particularly in critical aspects such as dynamic access control, privilege tiering, and data anonymization. On one hand, existing platforms lacked attribute-based encryption (ABE) enabled fine-grained access control strategies, impeding effective differentiation of data access privileges across distinct user roles; On the other hand, key technologies like differential privacy and federated learning remained immature in educational contexts, especially regarding systematic validation of privacy-enhancing techniques deployed at edge computing nodes (38, 40). These issues could trigger systemic risks including leakage of students’ sensitive information, misuse of physiological data, and reverse inference of behavioral models, substantially impeding the adoption of digital intelligence technologies in sports education.

##### 4.3.2.3 Imbalanced digital resource allocation

The successful implementation of digital-intelligent technologies in physical education depends heavily on teachers’ digital literacy, institutional infrastructure readiness, and support from the regional digital ecosystem. As a result, significant challenges persist in technological integration and pedagogical adaptation across diverse teaching environments (61). Effective application in physical education specifically requires high-performance hardware, stable network connectivity, and teachers’ autonomous operational competencies. However, substantial disparities in technological preparedness and resource accessibility exist across regions and teacher demographics. On one hand, experienced teachers often face difficulties in operating wearable devices, managing data interfaces, and navigating instructional software systems (62, 63). On the other hand, structural mismatches between the high cost of digital equipment (e.g., AR/VR platforms, motion capture systems) and the limited funding available in remote or K–12 school settings significantly impede scalable deployment (64). Moreover, the lack of standardization and transferability in digital instructional materials and motor skill visualization resources reduces the efficiency of instructional scenario reuse and limits content coverage, thereby undermining system scalability across different educational levels and contexts.

## 5 Discussion

This study systematically reviewed and analyzed the application of digital-intelligent technologies in physical education, yielding three key findings: (1) The adoption of digital-intelligent technologies in physical education has entered a phase of stable diffusion, characterized by stage-specific developmental trajectories; (2) Existing intelligent systems continue to face technical bottlenecks related to algorithmic adaptability, data processing efficiency, and pedagogical scenario modeling; (3) Future advancements should prioritize the development of teacher–student interactive systems and the establishment of flexible, adaptive governance frameworks to promote the deep integration of digital-intelligent technologies with educational goals. These findings not only illuminate the current state of development in the field but also provide a theoretical foundation for future roadmapping and the identification of critical implementation challenges.

### 5.1 Evolution of DIT applications in physical education

**Figure 3:**
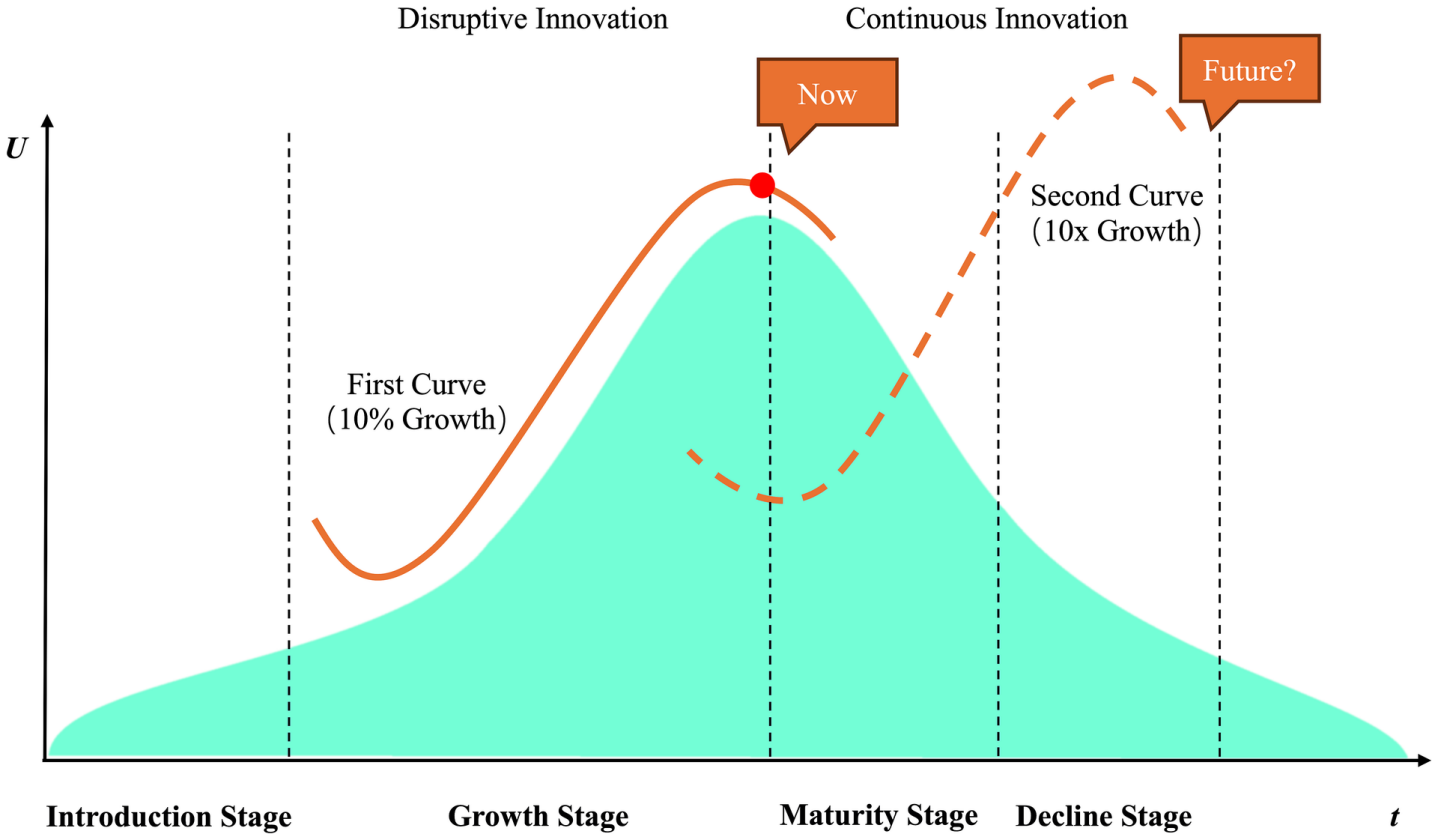
The evolutionary trends of DIT in the domain of physical education.

Consistent with the findings of Zhou, Wu et al. (7), tan integrated technological architecture—encompassing data-driven decision-making mechanisms, intelligent assistance systems, and IoT-enabled perception networks—is progressively reshaping the spatial configurations and pedagogical practices of physical education. This transformation has driven the field toward increased intelligence and personalization, exhibiting a distinct diffusion trajectory. Since 2022, coinciding with the shift of artificial intelligence into the “large-scale model training” phase, research on digital-intelligent technologies in physical education has experienced a significant surge, with 66 peer-reviewed empirical studies published within just 2 years. This expansion aligns closely with the S-curve model described in innovation diffusion theory (65) (see Figure 3), thereby validating the “critical mass” effect proposed by the theory. At present, the development of digital-intelligent technologies in physical education has entered a stabilized diffusion phase, characterized by increasing societal recognition and institutional integration, alongside continuous technological optimization and refinement.

Despite the increasing prominence of digital-intelligent technologies in education, substantial disparities persist in adoption levels across educational stages. Higher education institutions typically demonstrate more advanced technological integration, benefiting from superior resource availability and technical capacity, whereas primary and secondary education sectors remain comparatively underdeveloped. On March 15, 2022, the UK Department for Education released the report *Education Technology: Exploring Digital Maturity in Schools* (66), which revealed that the majority of K–12 schools in the UK were still in the early stages of digital maturity, with only 9% achieving advanced levels—highlighting the uneven progression of digital transformation across the education system. This phenomenon of “gradient diffusion” underscores the critical importance of institutional support, technological infrastructure, and teacher competency as key determinants of diffusion trajectories. As adoption approaches a plateau, the marginal value-added potential of digital technologies trends toward saturation. According to Handy’s *Second Curve Theory*, as technologies mature and proliferate, their growth potential asymptotically approaches diminishing returns. In this context, physical education must proactively pursue discontinuous innovation pathways to transition digital-intelligent technologies from simple tool integration toward holistic value re-engineering. Extensible and scalable technological capacities will thus become decisive variables in driving the next phase of deep digital transformation in education (67).

However, the proliferation of technology does not inherently guarantee meaningful educational outcomes. The *Global Education Monitoring Report* (68) emphasizes the “Technology on Our Terms” principle, advocating for the prioritization of appropriateness, equity, and sustainability in educational technology adoption. In resource-constrained regions, the deployment of digital-intelligent technologies often encounters financial limitations, infrastructural deficiencies, and pedagogical capacity gaps, resulting in pronounced structural inequities. Moreover, the adoption of such technologies may produce unintended negative consequences, including the erosion of core educational values and the emergence of new ethical dilemmas. For example, excessive dependence on data monitoring systems can undermine teacher–student interactions and hinder the development of students’ motor intuition. The collection of students’ physiological data raises serious privacy concerns, while algorithmic biases related to gender or physical constitution embedded in performance analytics risk reinforcing discriminatory practices in novel forms. These adverse factors directly undermine teachers’ perceived performance, social influence, and behavioral intention to adopt technology (69), thereby weakening the overall effectiveness of digital technology implementation and diffusion. Accordingly, there is an urgent need to strengthen educational systems’ adaptive regulatory capacities to ensure that digital-intelligent technologies are equitably accepted and responsibly integrated into physical education—serving the best interests of all students and ultimately realizing the vision of intelligent physical education.

### 5.2 Technological constraints of DIT in physical education

The current application of digital-intelligent technologies in physical education is undergoing a critical transition from traditional machine learning approaches to more advanced deep learning paradigms. This evolution fundamentally responds to the increasing demand for dynamic adaptability and personalized feedback in educational contexts. Now, the limitations of traditional machine learning algorithms have become increasingly evident, particularly when applied to complex, multimodal educational environments. (a) Traditional algorithms’ heavy reliance on manual feature engineering limits their ability to effectively process unstructured pedagogical data generated through teacher–student interactions (e.g., group movement trajectories), thereby constraining movement recognition dimensionality (57). (b) The lack of swarm intelligence technologies impedes the simulation of distributed cognitive processes in classroom environments, reducing the real-time responsiveness of pedagogical interventions. (c) Inadequate optimization algorithms result in low system stability when confronted with sensor signal drift or data loss, increasing the likelihood of misclassification and compromising the credibility of instructional analytics. This structural mismatch between algorithmic ecosystems and pedagogical scenario requirements reflects a “technology-first, education-lag” paradox, which constitutes a significant technical bottleneck hindering the scalability of DIT in basic education.

To address these challenges, next-generation explainable artificial intelligence (XAI) technologies are being developed to integrate multimodal perception, causal inference, federated learning (FL), and neuro-symbolic reasoning, aiming to construct more transparent, stable, and pedagogically aligned educational AI systems. Based on the synthesis of current research, XAI demonstrates strong potential to catalyze a shift in physical education from experience-based instruction to data-informed decision-making. By leveraging multimodal feedback, FL, and causal inference, XAI enables real-time visualization of instructional processes, enhances training attribution precision, and improves assessment reliability while ensuring data privacy and security (see Table 3).

**Table 3:** The technological framework for transparent AI decision-making in physical education.

Technology tool	Core function	Application scenario	Application example	Ref.
Multimodal Explanation	Action Analysis and Feedback Visualization	Enables comprehensive cross-modal analysis through the integration of visual data (motion capture video), sensor-derived data (wearable devices), and textual inputs (teaching reflections) to refine multimodal interpretation.	**a. Motion Correction and Performance Optimization:** AI-powered analysis assesses students’ basketball shooting mechanics, producing natural language feedback (e.g., “Insufficient arm angle”) and pinpointing key video frames to aid coaches and students in efficiently identifying and addressing movement inefficiencies. **b. Comprehensive Physical Fitness Evaluation:** By leveraging multi-source data fusion (e.g., heart rate monitoring, gait analysis), the system analyzes declines in physical fitness by identifying underlying contributing factors (e.g., “Fatigue accumulation resulting from consecutive high-intensity training”).	(93–99)
Explainable Reinforcement Learning (XRL)	Dynamic Optimization of Training Strategies	The system devises personalized training programs through AI-driven decision-making, dynamically adjusting to students’ real-time performance metrics while offering transparent justifications for its recommendations.	**a. Transparency in Training Strategy Adaptation:** Explainable Reinforcement Learning (XRL) improves the transparency of training strategy adaptation by providing explicit justifications for endurance training modifications (e.g., “Recent aerobic metabolism indicators failed to meet expected thresholds”). **b. Risk-Based Training Suspension Mechanism:** The system issues risk-based alerts by elucidating the rationale for suspending specific training activities (e.g., “Joint load surpasses the historical safety threshold”).	(100–103)
Federated Learning with Privacy Protection	Cross-institutional Data Collaboration	Educational institutions, families, community organizations, and sports clubs engage in collaborative sharing of training models while upholding stringent data privacy protections.	**a. Identification of Group-Specific Physical Fitness Trends:** Federated feature attribution facilitates the identification of shared physical fitness patterns among students across diverse regions (e.g., “Students in northern regions experience a more pronounced decline in flexibility during winter”) while safeguarding individual data privacy. **b. Distributed Performance Evaluation Mechanism:** Edge computing devices autonomously generate motion performance insights (e.g., “Improvements in jump power correlate with enhanced leg muscle activation”) and transmit only aggregated outcomes, thereby ensuring data security and minimizing exposure to centralized data repositories.	(104)
Causal Inference and Neuro-Symbolic Methods	Training Effect Attribution and Rule Integration	Minimizes misleading correlations by eliminating spurious associations (e.g., erroneously attributing performance outcomes to morning exercise time when sleep quality serves as the primary determinant).	**a. Causal Inference and Performance Evaluation:** Utilizes counterfactual validation to evaluate the efficacy of training methodologies (e.g., “A reduction in strength training frequency is projected to result in lower explosive power scores”). **b. Knowledge-Driven Model Optimization:** Incorporates sports science principles (e.g., “Maximal oxygen uptake is positively correlated with interval training duration”) into neural network models to derive expert-informed recommendations (e.g., “Increasing interval running volume directly enhances VO₂ max”).	(105, 106)

However, the successful deployment of XAI in physical education classrooms hinges on balancing model complexity with interpretability and ensuring alignment with authentic pedagogical tasks. Achieving this goal necessitates continuous algorithmic innovation, alongside interdisciplinary collaboration spanning sport science, pedagogy, and human–computer interaction.

### 5.3 Evolutionary trajectories of DIT in physical education

Digital-intelligent technologies integrate a range of cutting-edge innovations—including augmented/virtual reality (AR/VR), artificial intelligence (AI), and edge computing—to construct virtual embodied learning environments that transcend the spatial and perceptual limitations of traditional physical education. This integration has facilitated the initial realization of digital-intelligent transformation in the field. However, several critical constraints have surfaced that hinder further advancement: imbalances between technological supply and pedagogical demand, unresolved data ethics risks, and deficits in teachers’ digital competencies. These barriers, as summarized in Table 4, highlight the necessity of addressing both technical and contextual factors in the next stage of digital-intelligent development in physical education (see Table 4).

**Table 4:** Developmental paradoxes of DIT in physical education.

Type	Contradiction	Proposed solution	Ref.
Resource	The substantial investment required for the research and development of intelligent training systems stands in stark contrast to the fragmented distribution and limited accessibility of equipment in grassroots educational institutions.	**Policy Framework and Technological Accessibility:** Designate specialized funds for educational informatization to facilitate the development and deployment of intelligent training systems in grassroots educational institutions. Promote the integration of open-source technologies in education to mitigate technical barriers and improve accessibility.	(107–109)
Security	The inherent sensitivity of motion-related biological feature data presents significant challenges to data privacy and security, whereas the ongoing advancement of algorithms demands substantial data utilization for optimization.	**Differential Privacy and Blockchain-Based Traceability Mechanisms:** Desensitization of motion video data via key point skeleton extraction to safeguard individual privacy while retaining critical movement-related information. Blockchain-enabled immutable records of data usage to enhance transparency, strengthen security, and facilitate comprehensive auditability of data access.	(110, 111)
Evolution	The frequent monthly iterations of AI algorithms stand in stark contrast to the conventional annual training cycle of educators, resulting in a pronounced disparity in technological adaptation and pedagogical integration.	**Framework for Digital Literacy Certification:** An integrated learning analytics dashboard designed to monitor user progress and generate real-time insights into the development of digital competencies. A systematic evaluation framework for intelligent teaching tools to assess their pedagogical efficacy, usability, and alignment with instructional objectives.	(112–116)

**Figure 4:**
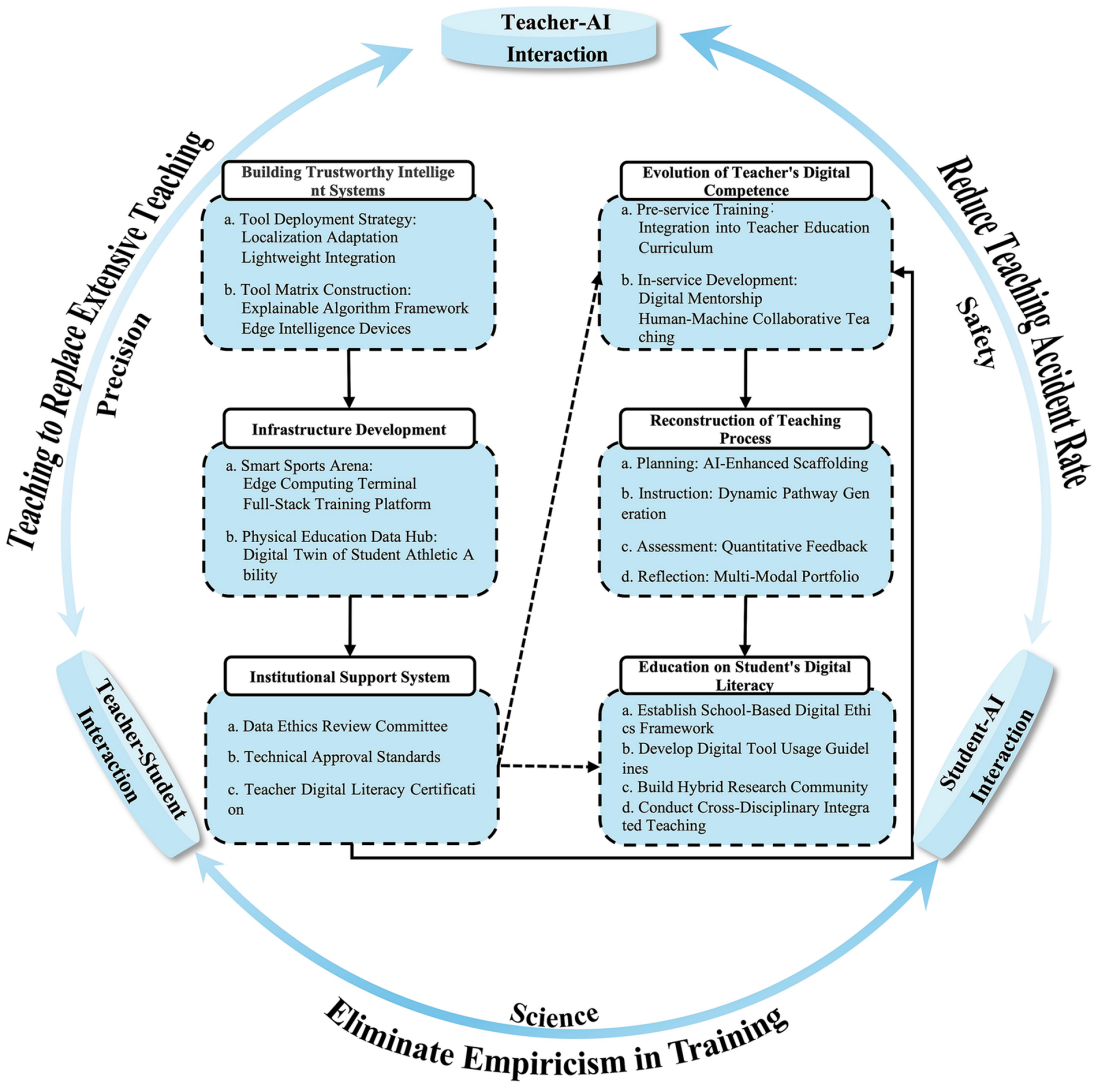
The triadic interaction system of “Teacher-DIT-Student”.

Amid the ongoing wave of educational digital transformation, the integration of digital-intelligent technologies into physical education demands not only technical adaptation but also alignment with kinesiological principles and pedagogical contextualization. Drawing on the developmental experiences of AI in education, this study proposes a Teacher–DIT–Student Interaction Framework (see Figure 4), guided by the principles of human-centered technological design. The framework comprises three core components: (a) Policy Safeguards: Establishing robust institutional mechanisms, including data ethics review boards, technology admissibility criteria, and teacher digital literacy certification systems, to ensure responsible and secure deployment of technologies. (b) Resource Infrastructure: Enhancing equitable resource allocation by constructing AI-enabled physical education environments and developing reliable intelligent systems customized for the unique demands of sports pedagogy. (c) Capacity Building: Embedding digital literacy development across the entire professional lifecycle of educators and reconfiguring human–AI collaborative pedagogical workflows to support adaptive instructional practices.

## 6 Conclusion and limitations

Through the systematic synthesis of empirical studies drawn from multidisciplinary academic databases, this review offers a comprehensive account of digital-intelligent technologies (DIT) applications in the physical education domain. The findings highlight three critical transformative dimensions: (1) motion data visualization, which enables real-time monitoring and feedback on performance; (2) pedagogical personalization, which allows instructional strategies to be tailored to learners’ individual characteristics; and (3) context-aware intelligence, which supports adaptive responses based on environmental cues and biometric data. Core technologies—including biosensors, virtual simulations, and adaptive learning platforms—are mitigating spatial–temporal constraints and redefining paradigms of embodied learning. To ensure that such technological advancements contribute substantively to holistic human development, the professional competencies of future physical educators must evolve along two principal dimensions: (a) pedagogical adaptation, entailing the effective incorporation of DIT tools into instructional design, and (b) scenario-based intelligence, which refers to the capacity to orchestrate collaborative learning environments involving human-AI interaction.

Despite its contributions, this systematic review is subject to several limitations. (1) Limited database coverage—While the review draws from major academic databases, relevant studies from non-indexed repositories or conference proceedings may have been excluded. (2) Methodological Heterogeneity—Variations in study design, technology types, and outcome indicators hindered advanced synthesis (e.g., meta-analysis). (3) Lack of theoretical integration—The review does not fully incorporate educational theory, particularly concepts of embodied pedagogy, which limits its explanatory depth. (4) Exclusion of gray literature—By prioritizing peer-reviewed sources, the review excludes theses and preprints, which may introduce publication bias and lead to an overrepresentation of studies with statistically significant findings. (5) Geographic concentration—Around 75% of the included studies were conducted in China, which may limit the generalizability of the findings to broader educational settings. (6) Design-related interpretive limitations—Although risk-of-bias was assessed using appropriate JBI tools, the predominance of observational and quasi-experimental designs—often lacking randomization or control conditions—may compromise the internal validity and robustness of the findings. These design-related risks were accounted for in the narrative synthesis but should be interpreted with caution.

## 7 Future directions

Future studies should address these limitations by incorporating gray literature, diversifying geographic data sources, and fostering interdisciplinary integration. In particular, bridging educational theory, sports science, and intelligent technology research is essential to facilitate a dialectical synthesis between technological innovation and pedagogical coherence.

## Data Availability

The original contributions presented in the study are included in the article/Supplementary material, further inquiries can be directed to the corresponding authors.
