# Supplementary material for: Application of digital-intelligent technologies in physical education: a systematic review
Source: Front Public Health. 2025 Jul 24;13:1626603. doi: 10.3389/fpubh.2025.1626603 (PMC12328460; doi:10.3389/fpubh.2025.1626603)
Supplement: Supplementary file 1 [file Data_Sheet_1.docx]

Supplementary Material

# Database Search Algorithms

1. Scopus search algorithm: 2213

(Used the following Filters: Humans, English, Article, all open to access)

(TITLE-ABS(physical education) OR TITLE-ABS(physical education teachers) OR TITLE-ABS(physical education teaching) OR TITLE-ABS(physical education class) OR TITLE-ABS(physical education curriculum) OR TITLE-ABS(school physical education) OR TITLE-ABS(sports study)) AND (TITLE-ABS(Artificial Intelligence) OR TITLE-ABS(AI) OR TITLE-ABS(Internet of Things) OR TITLE-ABS(intelligent sensor) OR TITLE-ABS(cloud computing) OR TITLE-ABS(Artificial Neural Network) OR TITLE-ABS(blockchain) OR TITLE-ABS(Sora) OR TITLE-ABS(Natural Language Processing) OR TITLE-ABS(Digital Technology) OR TITLE-ABS(ChatGPT) OR TITLE-ABS(Decision Tree) OR TITLE-ABS(Convolutional Neural Networks) OR TITLE-ABS(Recurrent Neural Network) OR TITLE-ABS(Naive Bayes model) OR TITLE-ABS(Computational Vision) OR TITLE-ABS(Voice recognition) OR TITLE-ABS(face recognition) OR TITLE-ABS(Deep Learning) OR TITLE-ABS(Machine Learning) OR TITLE-ABS(Logic programming) OR TITLE-ABS(fuzzy logic) OR TITLE-ABS(Ontology engineering) OR TITLE-ABS(Virtual Reality) OR TITLE-ABS(Augmented Reality) OR TITLE-ABS(Mixed Reality) OR TITLE-ABS(image processing) OR TITLE-ABS(Wearable smart devices) OR TITLE-ABS(Knowledge Graph) OR TITLE-ABS(Multimodal Models) OR TITLE-ABS(Situation Awareness) OR TITLE-ABS(Expert system) OR TITLE-ABS(chatbot robot) OR TITLE-ABS(intelligent tutoring system) OR TITLE-ABS(virtual agent) OR TITLE-ABS(conversational agent) OR TITLE-ABS(recommended system) OR TITLE-ABS(feedback system) OR TITLE-ABS(personalized learning) OR TITLE-ABS(adaptive learning) OR TITLE-ABS(prediction system) OR TITLE-ABS(student model) OR TITLE-ABS(learner model) OR TITLE-ABS(data mining) OR TITLE-ABS(learning analytics) OR TITLE-ABS(prediction model) OR TITLE-ABS(automated evaluation) OR TITLE-ABS(algorithm) OR TITLE-ABS(natural language processing)) AND ( LIMIT-TO ( DOCTYPE,"ar" ) ) AND ( LIMIT-TO ( LANGUAGE,"English" ) ) AND ( LIMIT-TO ( OA,"all" ) )

2. Web of Science search algorithm: 244

(Refined by the following: Document Types: Articles; Languages: English Open access)

TI=(“Artificial Intelligence” OR “AI” OR “Internet of Things” OR “intelligent sensor” OR “cloud computing” OR “Artificial Neural Network” OR “blockchain” OR “Sora” OR “Natural Language Processing” OR “Digital Technology” OR “ChatGPT” OR “Decision Tree” OR “Convolutional Neural Networks” OR “Recurrent Neural Network” OR “Naive Bayes model” OR “Computational Vision” OR “Voice recognition” OR “face recognition” OR “Deep Learning” OR “Machine Learning” OR “Logic programming” OR “fuzzy logic” OR “Ontology engineering” OR “Virtual Reality” OR “Augmented Reality” OR “Mixed Reality” OR “image processing” OR “Wearable smart devices” OR “Knowledge Graph” OR “Multimodal Models” OR “Situation Awareness” OR “Expert system” OR “chatbot robot” OR “intelligent tutoring system” OR “virtual agent” OR “conversational agent” OR “recommended system” OR “feedback system” OR “personalized learning” OR “adaptive learning” OR “prediction system” OR “student model” OR “learner model” OR “data mining” OR “learning analytics” OR “prediction")) AND TI=(“physical education” OR “physical education teachers” OR “physical education teaching” OR “physical education class” OR “physical education curriculum” OR “school physical education” OR “sports study”)) OR AB=(“Artificial Intelligence” OR “AI” OR “Internet of Things” OR “intelligent sensor” OR “cloud computing” OR “Artificial Neural Network” OR “blockchain” OR “Sora” OR “Natural Language Processing” OR “Digital Technology” OR “ChatGPT” OR “Decision Tree” OR “Convolutional Neural Networks” OR “Recurrent Neural Network” OR “Naive Bayes model” OR “Computational Vision” OR “Voice recognition” OR “face recognition” OR “Deep Learning” OR “Machine Learning” OR “Logic programming” OR “fuzzy logic” OR “Ontology engineering” OR “Virtual Reality” OR “Augmented Reality” OR “Mixed Reality” OR “image processing” OR “Wearable smart devices” OR “Knowledge Graph” OR “Multimodal Models” OR “Situation Awareness” OR “Expert system” OR “chatbot robot” OR “intelligent tutoring system” OR “virtual agent” OR “conversational agent” OR “recommended system” OR “feedback system” OR “personalized learning” OR “adaptive learning” OR “prediction system” OR “student model” OR “learner model” OR “data mining” OR “learning analytics” OR “prediction")) AND AB=(“physical education” OR “physical education teachers” OR “physical education teaching” OR “physical education class” OR “physical education curriculum” OR “school physical education” OR “sports study”) and Article (Document Types) and English (Languages) and Open Access

3. ACM search algorithm: 59

(Title:(("Artificial Intelligence" OR "AI" OR "Internet of Things" OR "intelligent sensor" OR "cloud computing" OR "Artificial Neural Network" OR "blockchain" OR "Sora" OR "Natural Language Processing" OR "Digital Technology" OR "ChatGPT" OR "Decision Tree" OR "Convolutional Neural Networks" OR "Recurrent Neural Network" OR "Naive Bayes model" OR "Computational Vision" OR "Voice recognition" OR "face recognition" OR "Deep Learning" OR "Machine Learning" OR "Logic programming" OR "fuzzy logic" OR "Ontology engineering" OR "Virtual Reality" OR "Augmented Reality" OR "Mixed Reality" OR "image processing" OR "Wearable smart devices" OR "Knowledge Graph" OR "Multimodal Models" OR "Situation Awareness" OR "Expert system" OR "chatbot robot" OR "intelligent tutoring system" OR "virtual agent" OR "conversational agent" OR "recommended system" OR "feedback system" OR "personalized learning" OR "adaptive learning" OR "prediction system" OR "student model" OR "learner model" OR "data mining" OR "learning analytics" OR "prediction model" OR "automated evaluation" OR "algorithm" OR "natural language processing") AND ("physical education" OR "physical education teachers" OR "physical education teaching" OR "physical education class" OR "physical education curriculum" OR "school physical education" OR "sports study"))) AND (Abstract:(("Artificial Intelligence" OR "AI" OR "Internet of Things" OR "intelligent sensor" OR "cloud computing" OR "Artificial Neural Network" OR "blockchain" OR "Sora" OR "Natural Language Processing" OR "Digital Technology" OR "ChatGPT" OR "Decision Tree" OR "Convolutional Neural Networks" OR "Recurrent Neural Network" OR "Naive Bayes model" OR "Computational Vision" OR "Voice recognition" OR "face recognition" OR "Deep Learning" OR "Machine Learning" OR "Logic programming" OR "fuzzy logic" OR "Ontology engineering" OR "Virtual Reality" OR "Augmented Reality" OR "Mixed Reality" OR "image processing" OR "Wearable smart devices" OR "Knowledge Graph" OR "Multimodal Models" OR "Situation Awareness" OR "Expert system" OR "chatbot robot" OR "intelligent tutoring system" OR "virtual agent" OR "conversational agent" OR "recommended system" OR "feedback system" OR "personalized learning" OR "adaptive learning" OR "prediction system" OR "student model" OR "learner model" OR "data mining" OR "learning analytics" OR "prediction model" OR "automated evaluation" OR "algorithm" OR "natural language processing") AND ("physical education" OR "physical education teachers" OR "physical education teaching" OR "physical education class" OR "physical education curriculum" OR "school physical education" OR "sports study")))

4. Taylor & Francis Online search algorithm: 31

(Refined by the following: Document Types: Articles; Languages: English Only show content I have full access to)

 [[Abstract: "artificial intelligence"] OR [Abstract: "ai"] OR [Abstract: "internet of things"] OR [Abstract: "intelligent sensor"] OR [Abstract: "cloud computing"] OR [Abstract: "artificial neural network"] OR [Abstract: "blockchain"] OR [Abstract: "sora"] OR [Abstract: "natural language processing"] OR [Abstract: "digital technology"] OR [Abstract: "chatgpt"] OR [Abstract: "decision tree"] OR [Abstract: "convolutional neural networks"] OR [Abstract: "recurrent neural network"] OR [Abstract: "naive bayes model"] OR [Abstract: "computational vision"] OR [Abstract: "voice recognition"] OR [Abstract: "face recognition"] OR [Abstract: "deep learning"] OR [Abstract: "machine learning"] OR [Abstract: "logic programming"] OR [Abstract: "fuzzy logic"] OR [Abstract: "ontology engineering"] OR [Abstract: "virtual reality"] OR [Abstract: "augmented reality"] OR [Abstract: "mixed reality"] OR [Abstract: "image processing"] OR [Abstract: "wearable smart devices"] OR [Abstract: "knowledge graph"] OR [Abstract: "multimodal models"] OR [Abstract: "situation awareness"] OR [Abstract: "expert system"] OR [Abstract: "chatbot robot"] OR [Abstract: "intelligent tutoring system"] OR [Abstract: "virtual agent"] OR [Abstract: "conversational agent"] OR [Abstract: "recommended system"] OR [Abstract: "feedback system"] OR [Abstract: "personalized learning"] OR [Abstract: "adaptive learning"] OR [Abstract: "prediction system"] OR [Abstract: "student model"] OR [Abstract: "learner model"] OR [Abstract: "data mining"] OR [Abstract: "learning analytics"] OR [Abstract: "prediction model"] OR [Abstract: "automated evaluation"] OR [Abstract: "algorithm"] OR [Abstract: "natural language processing"]] AND [[Abstract: "physical education"] OR [Abstract: "physical education teachers"] OR [Abstract: "physical education teaching"] OR [Abstract: "physical education class"] OR [Abstract: "physical education curriculum"] OR [Abstract: "school physical education"] OR [Abstract: "sports study"]] AND [Article Type: Article] AND [Language: English]

5. EBSCO search algorithm: 256

(Refined by the following: Scource Types: Academic Journals; Languages: English；full text；peer review)

TI ( (("Artificial Intelligence" OR "AI" OR "Internet of Things" OR "intelligent sensor" OR "cloud computing" OR "Artificial Neural Network" OR "blockchain" OR "Sora" OR "Natural Language Processing" OR "Digital Technology" OR "ChatGPT" OR "Decision Tree" OR "Convolutional Neural Networks" OR "Recurrent Neural Network" OR "Naive Bayes model" OR "Computational Vision" OR "Voice recognition" OR "face recognition" OR "Deep Learning" OR "Machine Learning" OR "Logic programming" OR "fuzzy logic" OR "Ontology engineering" OR "Virtual Reality" OR "Augmented Reality" OR "Mixed Reality" OR "image processing" OR "Wearable smart devices" OR "Knowledge Graph" OR "Multimodal Models" OR "Situation Awareness" OR "Expert system" OR "chatbot robot" OR "intelligent tutoring system" OR "virtual agent" OR "conversational agent" OR "recommended system" OR "feedback system" OR "personalized learning" OR "adaptive learning" OR "prediction system" OR "student model" OR "learner model" OR "data mining" OR "learning analytics" OR "prediction model" OR "automated evaluation" OR "algorithm" OR "natural language processing") AND ("physical education" OR "physical education teachers" OR "physical education teaching" OR "physical education class" OR "physical education curriculum" OR "school physical education" OR "sports study")) ) OR AB ( (("Artificial Intelligence" OR "AI" OR "Internet of Things" OR "intelligent sensor" OR "cloud computing" OR "Artificial Neural Network" OR "blockchain" OR "Sora" OR "Natural Language Processing" OR "Digital Technology" OR "ChatGPT" OR "Decision Tree" OR "Convolutional Neural Networks" OR "Recurrent Neural Network" OR "Naive Bayes model" OR "Computational Vision" OR "Voice recognition" OR "face recognition" OR "Deep Learning" OR "Machine Learning" OR "Logic programming" OR "fuzzy logic" OR "Ontology engineering" OR "Virtual Reality" OR "Augmented Reality" OR "Mixed Reality" OR "image processing" OR "Wearable smart devices" OR "Knowledge Graph" OR "Multimodal Models" OR "Situation Awareness" OR "Expert system" OR "chatbot robot" OR "intelligent tutoring system" OR "virtual agent" OR "conversational agent" OR "recommended system" OR "feedback system" OR "personalized learning" OR "adaptive learning" OR "prediction system" OR "student model" OR "learner model" OR "data mining" OR "learning analytics" OR "prediction model" OR "automated evaluation" OR "algorithm" OR "natural language processing") AND ("physical education" OR "physical education teachers" OR "physical education teaching" OR "physical education class" OR "physical education curriculum" OR "school physical education" OR "sports study")) )

6.Wiley Online Library search algorithm:142

(Used the following Filters: Open access content, journals)

“Artificial Intelligence” OR “AI” OR “Internet of Things” OR “intelligent sensor” OR “cloud computing” OR “Artificial Neural Network” OR “blockchain” OR “Sora” OR “Natural Language Processing” OR “Digital Technology” OR “ChatGPT” OR “Decision Tree” OR “Convolutional Neural Networks” OR “Recurrent Neural Network” OR “Naive Bayes model” OR “Computational Vision” OR “Voice recognition” OR “face recognition” OR “Deep Learning” OR “Machine Learning” OR “Logic programming” OR “fuzzy logic” OR “Ontology engineering” OR “Virtual Reality” OR “Augmented Reality” OR “Mixed Reality” OR “image processing” OR “Wearable smart devices” OR “Knowledge Graph” OR “Multimodal Models” OR “Situation Awareness” OR “Expert system” OR “chatbot robot” OR “intelligent tutoring system” OR “virtual agent” OR “conversational agent” OR “recommended system” OR “feedback system” OR “personalized learning” OR “adaptive learning” OR “prediction system” OR “student model” OR “learner model” OR “data mining” OR “learning analytics” OR “prediction" in Abstract and "“physical education” OR “physical education teachers” OR “physical education teaching” OR “physical education class” OR “physical education curriculum” OR “school physical education” OR “sports study”" in Abstract

# Supplementary Figures and Tables

## Supplementary Figures

**
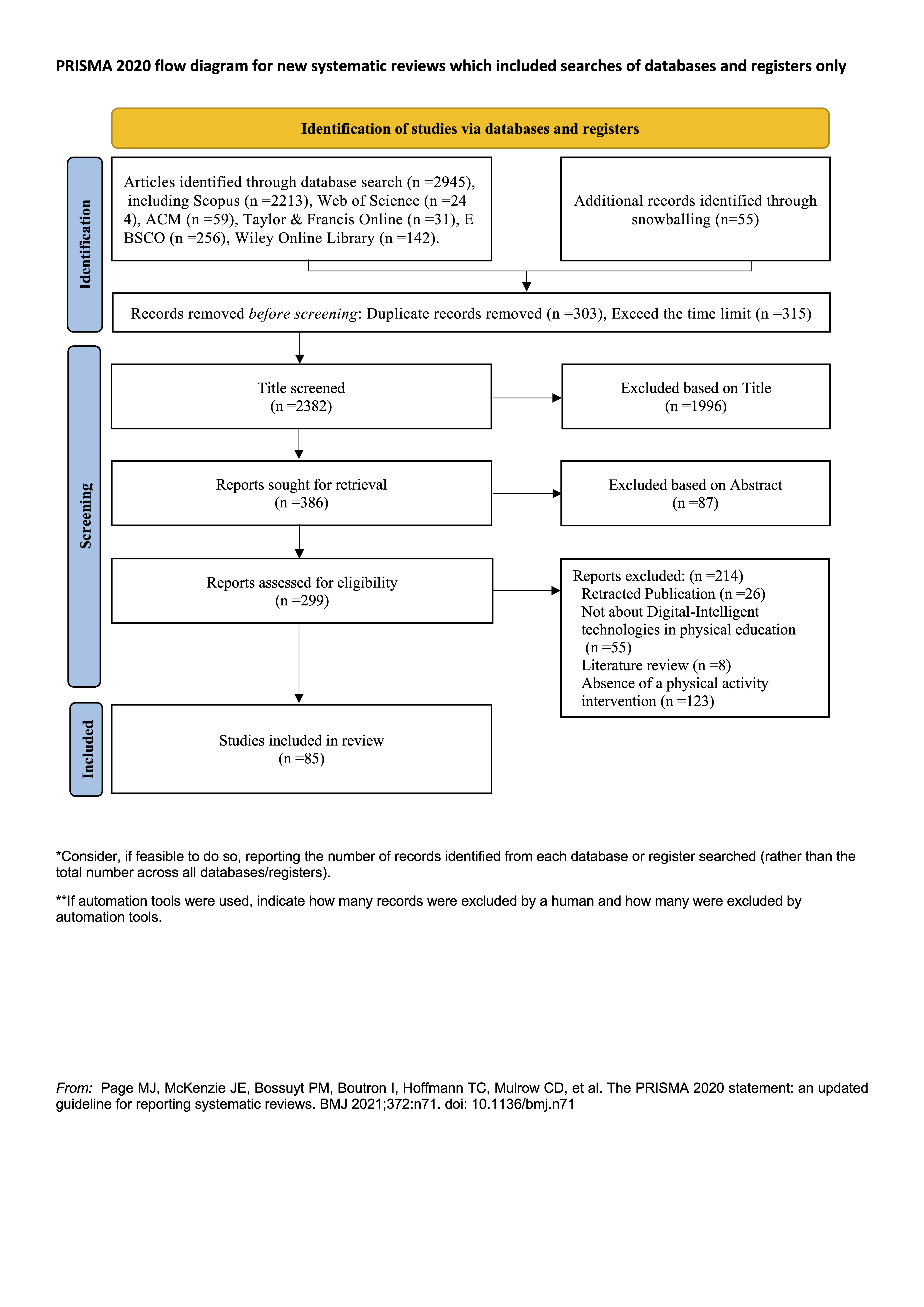
**

**Supplementary Figure 1.** PRISMA flow chart of selection process.


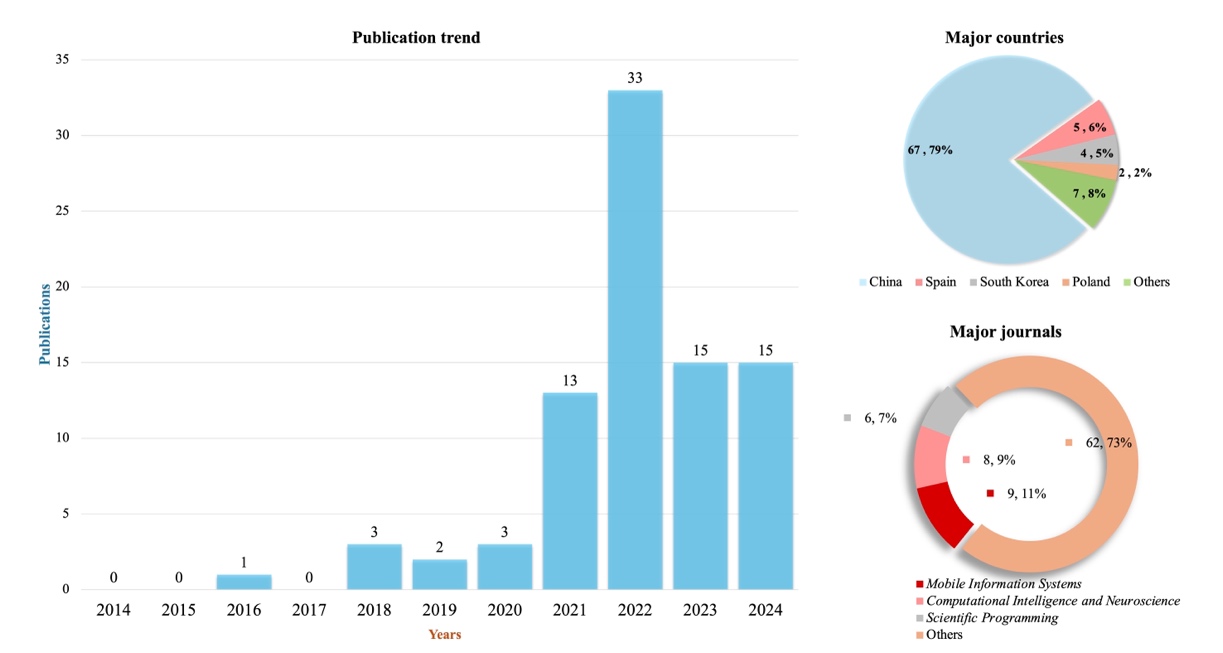


**Supplementary Figure 2.** The publication situation of empirical research on the DIT in Physical Education.


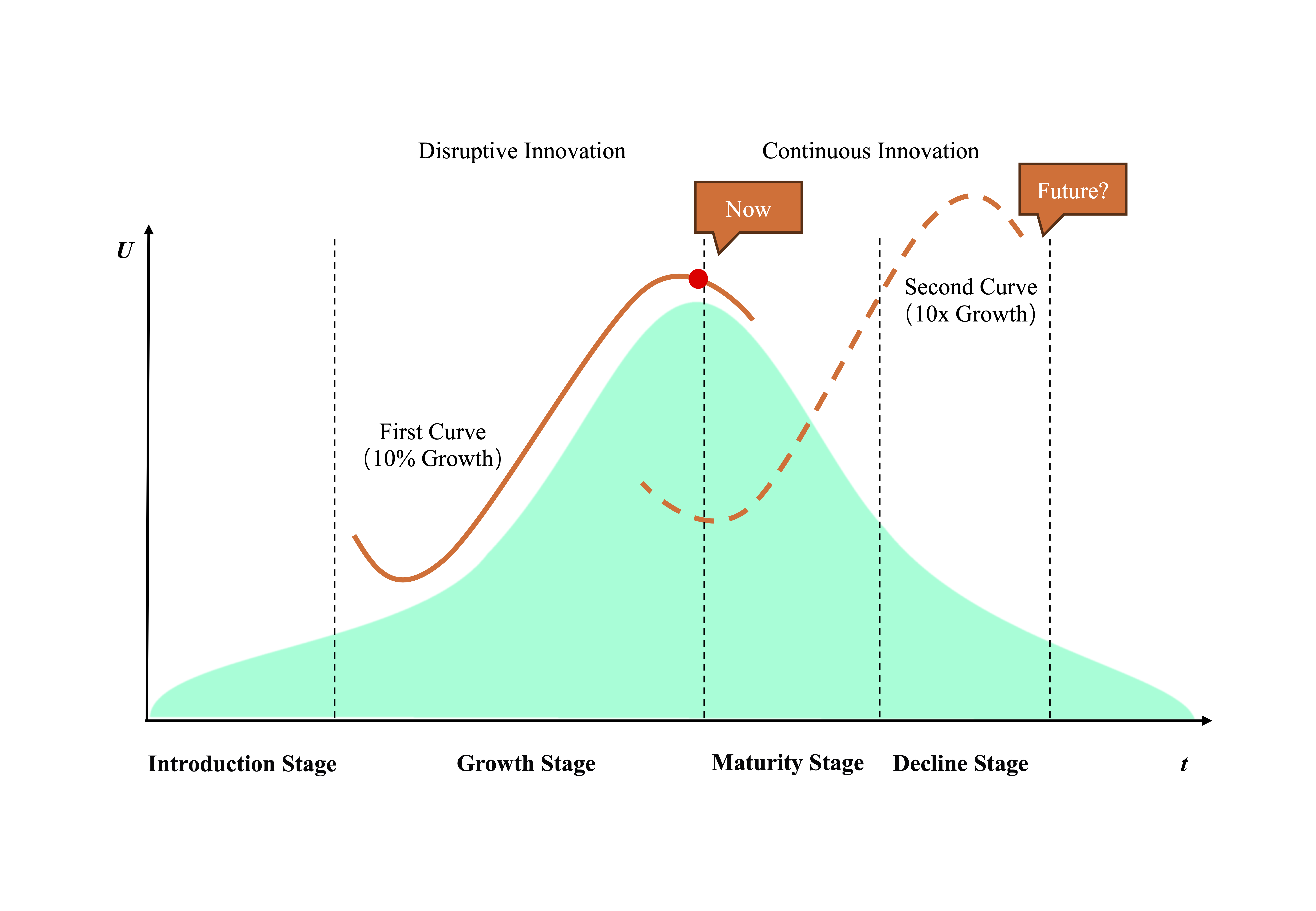


**Supplementary Figure 3.** The Evolutionary Trends of DIT in the Domain of Physical Education.


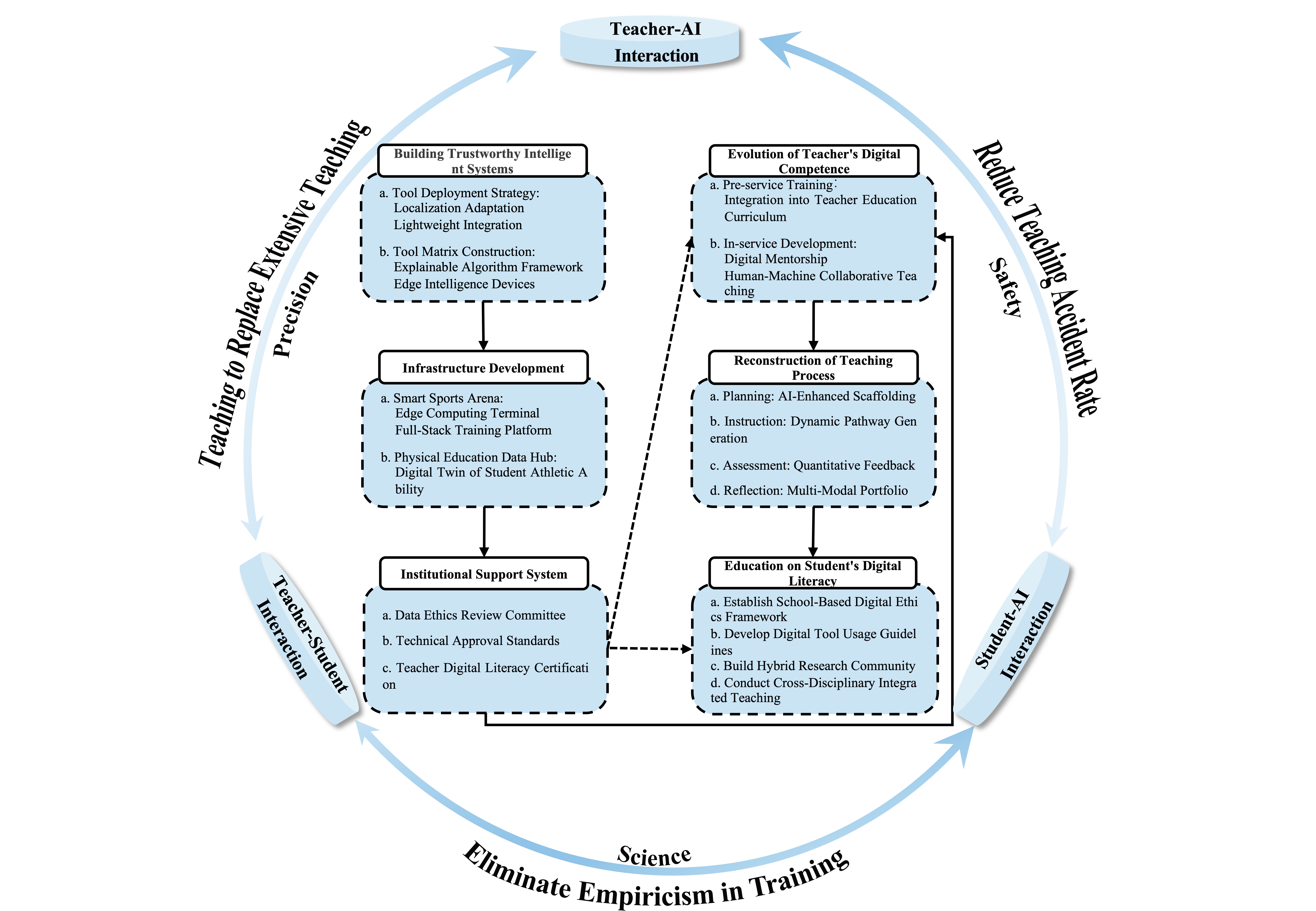


**Supplementary Figure 4.** The Triadic Interaction System of “Teacher-DIT-Student”.
